# Application of the optimized carbon monoxide rebreathing method for the measurement of total haemoglobin mass in chronic liver disease

**DOI:** 10.14814/phy2.14402

**Published:** 2020-03-24

**Authors:** James O.M. Plumb, James M. Otto, Shriya B. Kumar, Mark Wright, Walter Schmidt, Michael P.W. Grocott, Hugh E. Montgomery

**Affiliations:** ^1^ Respiratory and Critical Care Research Area NIHR Biomedical Research Centre University Hospital Southampton NHS Foundation Trust / University of Southampton Southampton UK; ^2^ Centre for Human Integrative Physiology Faculty of Medicine University of Southampton Southampton UK; ^3^ Anaesthesia, Perioperative Medicine and Critical Care Research Unit University Hospital Southampton NHSFT Southampton UK; ^4^ Shackleton Department of Anaesthesia University Hospital Southampton NHSFT Southampton UK; ^5^ University of Southampton Medical School Southampton UK; ^6^ Department of Hepatology University Hospital Southampton Southampton UK; ^7^ Department of Sports Medicine/Sports Physiology University of Bayreuth Bayreuth Germany; ^8^ Department of Anesthesiology Duke University School of Medicine Durham NC USA; ^9^ Centre for Human Health and Performance/ Institute of Sport, Exercise and Health University College London, and NIHR University College London Hospitals Biomedical Research Centre London UK

**Keywords:** anemia, chronic liver disease, optimized carbon monoxide rebreathing method (oCOR), total hemoglobin mass (tHb‐mass)

## Abstract

**Background:**

Anemia is common in liver cirrhosis. This generally infers a fall in total hemoglobin mass (tHb‐mass). However, hemoglobin concentration ([Hb]) may fall due to an expansion in plasma volume (PV). The “optimized carbon monoxide rebreathing method” (oCOR) measures tHb‐mass directly and PV (indirectly using hematocrit). It relies upon carboxyhemoglobin (COHb) distribution throughout the entire circulation. In healthy subjects, such distribution is complete within 6–8 min. Given the altered circulatory dynamics in cirrhosis, we sought in this pilot study, to assess whether this was true in cirrhosis. The primary aim was to ascertain if the standard timings for the oCOR were applicable to patients with chronic liver disease and cirrhosis. The secondary aim was to explore the applicability of standard CO dosing methodologies to this patient population.

**Methods:**

Sixteen patients with chronic liver parenchymal disease were studied. However, tHb‐mass was determined using the standard oCOR technique before elective paracentesis. Three subjects had an inadequate COHb% rise. In the remaining 13 (11 male), mean ± standard deviation (*SD*) age was 52 ± 13.8 years, body mass 79.1 ± 11.4 kg, height 175 ± 6.8 cm. To these, mean ± *SD* dose of carbon monoxide (CO) gas administered was 0.73 ± 0.13 ml/kg COHb values at baseline, 6 and 8 min (and “7‐min value”) were compared to those at 10, 12, 15 and 20 min after CO rebreathing.

**Results:**

The “7‐min value” for median COHb% (IQR) of 6.30% (6.21%–7.47%) did not differ significantly from those at subsequent time points (8 min: 6.30% (6.21%–7.47%), 10 min: 6.33% (6.00%–7.50%), 12 min: 6.33% (5.90%–7.40%), 15 min: 6.37% (5.80%–7.33%), 20 min: 6.27% (5.70%–7.20%)). Mean difference in calculated tHb‐mass between minute 7 and minute 20 was only 4.1 g, or 0.6%, *p = *.68. No subjects reported any adverse effects.

**Conclusions:**

The oCOR method can be safely used to measure tHb‐mass in patients with chronic liver disease and ascites, without adjustment of blood sample timings. Further work might refine and validate appropriate dosing regimens.

## Graphical abstract

Anaemia is common in liver cirrhosis. This generally infers a fall in total haemoglobin mass (tHb‐mass). The “optimized carbon monoxide rebreathing method” (oCOR) measures tHb‐mass and PV. It relies upon carboxyhaemoglobin (COHb) distribution throughout the entire circulation. In healthy subjects, such distribution is complete within 6–8 min. Given the altered circulatory dynamics in cirrhosis, we sought to assess whether this was true in cirrhosis. The “7‐min value” for median COHb% (IQR) of 6.30% (6.21%–7.47%) did not differ significantly from those at subsequent time points. Therefore, the oCOR method can be safely used to measure tHb‐mass in patients with chronic liver disease and ascites, without adjustment of blood sample timings.

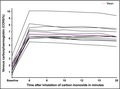

## 1 INTRODUCTION

The concentration of hemoglobin in the circulation ([Hb]) is determined by its total circulating mass (tHb‐mass) and the plasma volume (PV) in which it is suspended. Anemia, defined as a reduction in [Hb] to <120 g/l in nonpregnant females and <130 g/l in males (WHO, 1968), is common in patients with chronic liver disease (CLD), with a reported prevalence of 75% (Gonzalez‐Casas, Jones, & Moreno‐Otero, 2009). To this, a fall in tHb‐mass (due to gastrointestinal blood loss, hematinic deficiency, hemolysis, and bone marrow suppression) may contribute (Gkamprela, Deutsch, & Pectasides, 2017). So, too, may a rise in PV (Otto, Plumb, Clissold, et al., 2017), although this element is often ignored in clinical practice, largely due to difficulties in measuring PV.

The tHb‐mass can be measured directly by the methods of Burge and Skinner (Burge & Skinner, 1995), modified by Schmidt and Prommer (the optimized carbon monoxide (CO) rebreathing method (oCOR)) (Schmidt & Prommer, 2005) (see methods and Appendix 1 & 2). Here, binding of a known volume of CO to Hb, and measurement of carboxyhemoglobin concentration (COHb) allows tHb‐mass to be measured and, using this and knowing hematocrit, PV to be derived. Predominantly applied to monitor athletes’ responses to altitude training (Gore et al., 2013), it has more recently been used in clinical medicine (Ahlgrim et al., 2018; Diaz‐Canestro, Haider, Lundby, & Montero, 2019; Koponen et al., 2013; Otto, Plumb, Clissold, et al., 2017; Otto, Plumb, Wakeham, et al., 2017; Wrobel, Pottgiesser, Birkner, Deibert, & Ahlgrim, 2016). Data regarding oCOR application to CLD patients were limited (Wrobel et al., 2016), but we demonstrated a poor relationship between [Hb] and tHb‐mass in CLD (*r = *.410, *p = *.11) with PV explaining much of the variance in [Hb] (Otto, Plumb, Clissold, et al., 2017).

The accuracy of the oCOR technique depends on the completeness of COHb mixing in the circulation after CO inhalation (Garvican et al., 2010; Gore, Hopkins, & Burge, 2005) and this appears complete by 8 min (Gore et al., 2013; Schmidt & Prommer, 2005). As such, measurement of COHb% at 6 and 8 min (averaged to yield the “7 min” value) is commended (Schmidt & Prommer, 2005), and was used in our study. However, mixing is delayed in disease states such as cardiac failure (Ahlgrim et al., 2018) and polycythemia (Wachsmuth, Soria, Jimenez, & Schmidt, 2019). Admixture might be slowed down in CLD due to changes in regional (e.g. enteric) and global blood flow dynamics (Drazen et al., 2010; Iwakiri & Groszmann, 2006; Mayr et al., 2018; Murray, Dawson, & Sherlock, 1958; Scheinfeld, Bilali, & Koenigsberg, 2009; Takahashi et al., 2014), and due to the presence of expanded arteriovenous shunts (Schrier, 1988).

Meanwhile, the applicability of the standard oCOR dosing regimen has not been reported in CLD patients. The desired absolute rise in COHb from baseline‐ 4.0%–6.5% (ΔCOHb%) with peak levels <10% (Naef, Steiner, & Wehrlin, 2014; Plumb et al., 2018; Turner, Pringle, et al., 2014) represents a tradeoff between precision and safety. Cirrhosis leads to higher baseline COHb% due to elevated levels of inducible heme oxygenase (HO‐1) driving increased heme conversion to biliverdin, with associated breakdown of CO production (Iwakiri & Groszmann, 2006). Because most hemoximeters estimate COHb% to only a single decimal place, resulting in smaller increments in COHb% could result in lower precision of measurement (Turner, Pringle, et al., 2014; Turner, Richardson, Maxwell, & Pringle, 2014b). Meanwhile, COHb > 15% can be associated with headaches and changes in visual‐evoked potentials (Stewart, 1975).

Traditional oCOR methodology distinguishes between “trained” (athlete) and “untrained” (nonathlete, but healthy) subjects with 1.0 and 0.8 ml/kg doses of CO, respectively, being safely used for men and 0.7 and 0.6 ml/kg doses for women (Schmidt & Prommer, 2005). Of note, CLD patients are likely to be significantly more deconditioned than the “untrained” group in the original paper (Schmidt & Prommer, 2005) and, as such, further dose reduction might be indicated. “Appropriate dose reduction” has also been commended “for patients with significant anemia and or/morbid obesity” although these states were not clearly defined (Gore et al., 2005; Turner, Pringle, et al., 2014). Further, when body mass index is >30 kg m^2^, the use of ideal body weight (IBW) is advocated when calculating the CO dose to be administered (Wachsmuth et al., 2019). Such elements pose problems for the study of CLD patients. Firstly, up to three quarters may be anemic (Gonzalez‐Casas et al., 2009) which, if due to low tHb‐mass, could suggest the need for a lower CO dose. The fact that [Hb] relates poorly to tHb‐mass in CLD (Otto, Plumb, Clissold, et al., 2017) further confounds CO dose‐estimation. Finally, ascites and/or edema may confound measured body weight and prevalent sarcopenic and/or low body fat mass confound “ideal body weight.” The potential for delivering COHb% values beyond those desirable is therefore high.

In this prospective, pilot observational clinical study, we sought to address both of these issues. We sought primarily to ascertain if adequate circulatory mixing of CO occurs within the standard time frames (Schmidt & Prommer, 2005). Our secondary aim was to explore the applicability of standard CO dosing methodologies to this patient population.

## 2 MATERIALS AND METHODS

The study was conducted at the University Hospital Southampton NHS Foundation Trust between May 2015 and October 2017. Ethical approval was granted by the London—Camden and Kings Cross Research Ethics Committee (REC reference: 13/LO/1902), with an amendment allowing venous sampling for up to 20 min after CO inhalation being likewise approved. Written informed consent was obtained from all participants.

### 2.1 Subjects

Sixteen patients with CLD and diuretic‐refractory ascites were recruited by the clinical team from those undergoing elective day‐case paracentesis. We have previously published data derived from venous samples at 6 and 8 min after CO inhalation in 16 subjects (Otto, Plumb, Clissold, et al., 2017). In 5 of these, there was no venous sampling after 8 min. We thus obtained full data (venous sampling at 6 & 8 min then additionally at 10, 12, 15, and 20 min after CO inhalation) in an additional 5 patients, and report these data for the total of 16 patients (Table 1).

**Table 1:** Hemoglobin carbon monoxide measures

Patient number	[Hb] (g/l)	tHb‐mass (g)	CO “dose given” (ml)	CO dose (ml CO/kg body weight)	Absolute change in COHb% (pre‐CO exposure and 7 min later)
1*	98	396	50	0.6	7.2
2	104	628	48	0.5	4.5
3	105	431	44	0.6	5.5
4	117	664	64	0.8	5.8
5*	113	581	40	0.7	4.0
6	93	662	64	0.7	5.9
7	132	590	52	0.8	4.5
8	101	518	46	0.6	5.0
9	144	883	60	0.7	4.2
10^$^	184	799	64	1.0	4.9
11	100	648	72	0.8	6.7
12	118	787	70	0.9	5.5
13	121	828	70	0.8	4.7
*M* ± *SD*. Aside from [Hb] Median [IQR]	113.0 [100.5–126.5]	647.0 ± 148.9	57.23 ± 11.00	0.73 ± 0.14	5.20% ± 0.96%

* Female patient

^$^ Patient has polycythaemia rubra vera (PCV) and requires regular bloodletting for treatment CO (carbon monoxide); [Hb] (concentration of hemoglobin in venous blood); COHb% (percentage of hemoglobin in the form of carboxyhemoglobin).

### 2.2 Optimized carbon monoxide re‐breathing method (oCOR)

Here tHb‐mass was determined by oCOR on the morning immediately prior to abdominal paracentesis (Schmidt & Prommer, 2005) (see also Appendix 1 & 2). Venous COHb% was measured before and after 2 min rebreathing a known CO volume (0.5 to 1.0 ml/kg, with dose determined according to fitness, frailty, gender and [Hb] as described below). We sought a minimum ΔCOHb% of 4.0% to avoid inaccuracies in tHb‐mass measurements (see introduction).

The tHb‐mass was calculated using the difference in COHb% in blood samples obtained before and (averaged) 6 and 8 min after the inhalation of the CO bolus, and using blood samples drawn between 8 and 20 min. For these calculations, CO diffusion to myoglobin and CO exhaled until the respective time point was considered as previously published (Prommer & Schmidt, 2007) (e.g. in patients studied for 20 min, CO volume diffused to myoglobin ranged from 1.4 to 2.9 ml, and exhaled CO volume between 1.0 and 5.1 ml). For detailed calculation of tHb‐mass and blood volume derivatives see Appendix 1 & 2 and for further explanation see (Prommer & Schmidt, 2007).

### 2.3 Dosing of carbon monoxide

Carbon monoxide dosing aims to achieve an absolute ΔCOHb% of between 4.0% and 6.5%, between 6 and 8 min (the mean being referred to as the “7 min value”) after CO administration. We assumed that all the CLD patients were further debilitated and deconditioned beyond being simply “untrained” (see above), and thus used doses of 0.8 and 0.6 ml/kg CO for men and women, respectively. Two patients did not appear debilitated (World Health Organization performance status zero) (West & Jin, 2015), one of whom also who had polycythemia rubra vera (PRV) with a [Hb] of 184 g/l and dosing was increased to 0.9 and 1.0 ml/kg, respectively, in these.

It is advocated that consideration be given to further dose reduction in anemic subjects (<130 g/l in men, and <120 g/l in women (WHO, 1968)), and we followed this approach. In total, CO dose was reduced from the 0.8 or 0.6 ml/kg recommended for “untrained subjects” in 5 individuals (subjects 2, 3, 6, 8 & 9, Table 1) due to them being anemic (5 subjects, see Table 1) and then further reduced if their performance status was low (high number on the Zubrod scale) at the discretion of the investigators. This occurred in 3 subjects (2, 3 & 8 Table 1). If a subject's BMI is >30 kg m^2^, it is suggested that CO dosing is based on IBW (see introduction). However, this did not apply to any of our subjects.

### 2.4 Statistical analysis

Statistical analysis was performed using GraphPad prism version 7.0c for Apple Macintosh. The Shapiro–Wilk test for normal distribution was used. Values are presented as mean ± standard deviation (*SD*), unless otherwise stated. Median and interquartile range (IQR) are reported when variables were not normally distributed. Categorical variables are presented as frequency (%). Paired *t* tests were used to calculate differences in tHb‐mass at different time points. The Mann–Whitney U test was used to calculate differences in COHb% at different time points. Repeated measures ANOVA was used to compare calculated tHb‐mass data for each time point.

## 3 RESULTS

### 3.1 Carbon monoxide dosing

The mean ± *SD* CO dose administered was 0.73 ± 0.13 ml/kg (range 42–76 ml), with weight‐indexed doses ranging from 0.5–1.0 ml/kg. Mean dose to males was 0.75 ± 0.14 ml/kg, and to females 0.65 ± 0.07 ml/kg.

In three subjects, ΔCOHb% was <4% (2.6%, 2.7% and 3.2%). Their demographics are shown in Table 2. Two were women, being reflected in their somewhat lower heights and weights. However, no variables differed significantly from the 13 subjects in whom ΔCOHb% was sufficient to allow valid calculation of tHb‐mass (*p* = .08 for BMI, *p* = .46 for [Hb]). The doses received were in accordance with the description in the methods with the two females receiving 0.6 ml/kg, and the one male (who was anemic [Hb] 105 g/l), receiving 0.7 ml/kg). In the remaining 13 subjects, venous COHb% increased from a mean of 1.62% ± 0.77% at baseline to a median value of 6.3 (IQR 6.21–7.47) at “7 min” giving a mean ΔCOHb% of 5.26% ± 096% (range 4.0%–7.2%). Table 1 shows the doses administered and the ΔCOHb% for each patient, the latter being adequate for all 13 subjects (Table 1).

**Table 2:** Demographics of the three excluded patients

Patient	Age (years)	Gender	Height (cm)	Weight (kg)	BMI (kg m^2^)	[Hb] (g l)	CO dose in ml	ΔCOHb%
1	49	M	176	65.7	21.2	105	46	3.2
2	50	F	162	57.8	22.0	128	36	2.6
3	50	F	165.5	66.3	24.2	129	40	2.3

Note

The demographic details of the three subjects in whom the rise in COHb% was < 4%.

One patient had a peak COHb% of 10.1% after 6 min, exceeding that which most investigators would aim for. They suffered no ill effects, however, and their COHb% level was below 10% at 12 min.

The tHb‐mass could not be reliably calculated for the 3 patients in whom ΔCOHb% was < 4%. This left 13 patients (11 male; mean 52 ± 13.8 years, body mass 79.1 kg ± 11.4 kg, height 175 ± 6.8 cm, body mass index 25.8 ± 3.0 kg m^2^) for the final analysis. All had established CLD: nine alcoholic cirrhosis (one of whom also suffered hepatocellular carcinoma), one cryptogenic cirrhosis, one with chronic hepatitis C, one Budd–Chiari Syndrome and one nonalcoholic steatohepatitis.

In the 13 in whom COHb rise was > 4% (absolute value), median [Hb] was 113 g/l (IQR 101–127 g/l), mean ± *SD* tHb‐mass 647.3 g ± 148.9 g, PV 4221 ml ± 104 and blood volume 6,101 ± 1,194 ml. Ten patients (77%) were anemic (mean ± *SD* [Hb] 107 ± 9.6 g/l versus. 153.3 ± 27.2 g/l for those not anemic) (Table 1 and Appendix 1).

### 3.2 Carbon monoxide wash‐in curve

**Figure 1:**
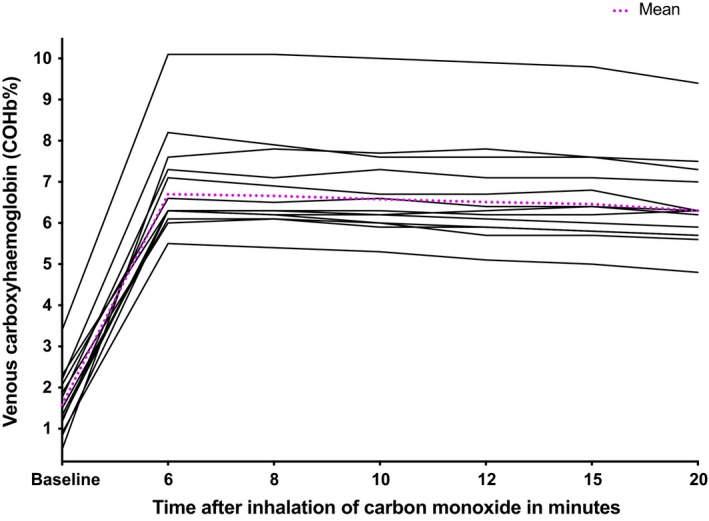
Carboxyhaemoglobin (COHb%) wash in curves from baseline to 20 minutes after CO re‐breathing. Each line represents one individual. Venous carboxyhaemoglobin samples taken at baseline to 20 minutes post inhalation of carbon monoxide gas.

**Figure 2:**
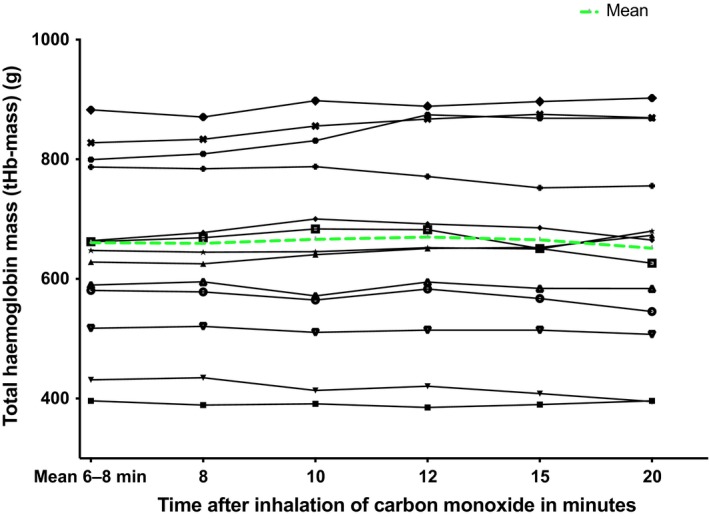
tHb‐mass data calculated for different COHb% sampling time points. Each line represents one individual. tHb‐mass was calculated using COHb% values in blood drawn from 6 different time points after inhalation of the carbon monoxide bolus.

Figure 1 shows the COHb% values from baseline to 20 min after CO inhalation. The peak occurred at either 6 or 8 min after inhalation (median 6.33% (IQR 6.25%–7.46%) and 6.30% (IQR 6.20%–7.47%) respectively. The values were 6.30% (6.21–7.47) at seven minutes, 6.33% (6.00%–7.50%) at 10 min, 6.33% (5.90%–7.40%) at 12 min, and 6.37% (5.80%–7.33%) at 15 min, The value at 7 min did not differ from that 20 min after CO inhalation (6.27% (5.70%–7.20%), *p* = .20). In keeping, calculated tHb‐mass was also near‐constant over this time period (mean ± *SD* being 647.3 ± 149.1 g at 6 min, 648.6 ± 149.1 g at 8 min, and 653.4 ± 162.0 g, 659.7 ± 163.2 g, 653.5 ± 164.9 g and 651.4 ± 167.9 g at 10, 12, 15, and 20 min, respectively (Figure 2)). The calculated tHb‐mass at minutes 7 and 20 were similar (*p = *.68) and differed by only 4.1g, or 0.6%, while repeated measures ANOVA confirmed no difference in tHb‐mass between any other timepoints (*p = *.48).

## 4 DISCUSSION

We have clarified, for the first time that the optimized carbon monoxide rebreathing method can be used to calculate tHb‐mass in patients with chronic liver disease without modification of the timing of venous sampling. Thus, COHb% values at 6 and 8 min giving a “7 min” (the mean of minutes 6 and 8) do not fall significantly over the next 12 min, and calculated tHb‐mass thus does not differ significantly (*p = *.68). This suggests that the mixing of CO in the blood is complete by “7 min” after inhaling the CO‐bolus, and that any blood sample between min 7 and min 20 can be used for the calculation of tHb‐mass (Figures 1 and 2). While some COHb% values appear to still be rising between minutes 6 and 8, this difference has little impact on calculated tHb‐mass. For example, calculated tHb‐mass in patient 2 (see Table 1) is 396.1 g versus 389 g for “minute 7” and 8, respectively.

These data suggest that any changes in COHb circulatory mixing kinetics in CLD do not necessitate adjustment of standard sample timings (Schmidt & Prommer, 2005), perhaps in contrast to the situation in heart failure (left ventricular ejection fraction < 30%) patients (Ahlgrim et al., 2018) and polycythemia (Wachsmuth et al., 2019).

For precision in tHb‐mass measurement by oCOR, the rise in carboxyhemoglobin (ΔCOHb%) is variably required to exceed an absolute value of 5% (typically 0.5%–1.5% at rest rising to 5%–7% post‐CO rebreathing) (Schmidt & Prommer, 2005; Turner, Richardson, et al., 2014), or to reach values of 5.5%–6% (Gore et al., 2005). Our own unpublished data suggest that values over 4.0% are essential for adequate reliability. Low doses (0.6 ml/kg) result in a ΔCOHb% of only 3.4 ± 0.4%, but yield similar tHb‐mass values to those when using 1.0 ml/kg (791 versus 788 g, respectively)‐ suggesting that a ΔCOHb% of <4% may be methodologically acceptable (Turner, Pringle, et al., 2014). In healthy subjects, a dose of 1 ml/kg has been shown to be adequate and safe (Turner, Pringle, et al., 2014). We have safely and effectively used 0.4–1 ml/kg in patients (Otto, Plumb, Clissold, et al., 2017; Otto, Plumb, Wakeham, et al., 2017), and the only other clinical study to provide data (in patients with coronary artery disease) used doses of 0.7 ml/kg for women and 0.8 ml/kg for men (Karlsen, Leinan, Aamot, Dalen, & Stoylen, 2016) and yielded a mean ΔCOHb% of 4.5%. Recent work in polycythemia subjects used 1.7 ml/kg and yielded an approximate ΔCOHb% of 3.7%. (Wachsmuth et al., 2019). Unfortunately other clinical studies did not report the doses used (Koponen et al., 2013; Wrobel et al., 2016). We accept that we cannot comment on accuracy based on these data as multiple measures at differing doses have not been taken. Further clinical research might address this question.

We show, however, that CO‐dosing to achieve ΔCOHb% values > 4% (with peaks < 10%) can be problematic in CLD patients. Mean CO doses were 0.75 and 0.65 ml/kg for males and females respectively, but ranged from 0.5–1 ml/kg (Table 1). Primary dosing was based on gender with the basic assumption that all participants were “untrained” by nature of their CLD. The original recommended doses of 0.8 or 0.6 ml for men and women, respectively, were used for dosing. However, only 36% (4 subjects) of the male participants in this study received 0.8 ml/kg. In 5 subjects, the dose was reduced as described above (Table 1). In the two male patients with a [Hb] ≤100 g/l one received a CO dose of 0.7 ml/kg and the other a dose of 0.6 ml/kg (yielding a ΔCOHb% of 5.9% and 7.2% respectively). Two male patients received higher doses (one patient with JAK2 positive polycythemia rubra vera (PRV) receiving 1 ml/kg) the other was young, unusually had no other comorbidities, had a very good exercise tolerance and was deemed “relatively fit” compared to the other subjects.

Using the recommended dosing for “untrained subjects” led to the exclusion of three patients due to “under‐dosing” (rise in COHb% of 2.6%, 2.7% and 3.2% respectively). Of note, two of these cases were women, one of whom had a body mass (57.8kg) lower than many other subjects‐ and who therefore only received 36 ml of CO gas. The remaining subject was a male who received 0.7 ml/kg CO dosing. It is possible that the calculated CO bolus will be too high or too low due to [Hb] not always correlating with tHb‐mass. By way of example, in the three most anemic patients ([Hb] 100, 98, and 93 g/L), tHb‐masses were 647.6 g for the person with [Hb] 100 g/l but 396 g/l 1 g in the person with [Hb] only 2 g/l lower ([Hb] 98 g/l—with the highest tHb‐mass of the three (662.3 g) being in the most anemic patient ([Hb] 93 g/l). In the 3 patients excluded from the original 16, this was the case: absolute ΔCOHb% values were 3.2%, 2.7% and 2.3%, respectively, making calculation of tHb‐mass less reliable. They did not have any documented CO gas leaks or other analytical errors that could have resulted in a low ΔCOHb%. In the case of a dilutional anemia resulting in a falsely low calculated CO bolus and subsequent low ΔCOHb% (<4%), it is possible to repeat the test within only a few hours (Naef et al., 2014; Plumb et al., 2018). In our previously published work relating to CLD patients, one subject with a [Hb] of only 69 g/l was given a dose of 0.4 ml/kg producing a ΔCOHb% of 4.7% (Otto, Plumb, Clissold, et al., 2017). This highlights the unpredictability of response in severely anemic patients to bolus CO administration—perhaps because [Hb] and tHb‐mass values are divorced when plasma volume is known to be deranged (Otto, Plumb, Clissold, et al., 2017).

Strengths of this study include the fact that the same two operators performed all oCOR tests in the same laboratory, using the same equipment thus reducing measurement error. Conditions were identical for all of the patients and all measurements were made prior to paracentesis.

Limitations include that fact that we did not objectively quantify “fitness.” Nor did we evaluate intersubject reliability by performing multiple tests on each patient. While theoretically possible that areas with very poor perfusion may not have equilibrated by 20 min (our last sample time), there was no appreciable rise in COHb% after the initial peak at either 6 or 8 min, making this unlikely, and any possible impact small. While accuracy might have been improved, we were reluctant to use higher CO doses due to the high prevalence of anemia (77%), and because no other reports exist of the use of the oCOR method in such patients. Due to the pilot nature of this work, we cannot comment specifically on safety but it is possible that, in future, doses could be adjusted according to baseline values, such that excessively high COHb% levels are avoided. However, it is important to recognize that “safe levels” of COHb are not exclusively about peak COHb%, and that the duration of raised COHb% is also important. Carboxyhemoglobin levels have been elevated to 20% in healthy volunteer studies without reported ill effects (González‐Alonso, Richardson, & Saltin, 2001).

## 5 FUTURE RESEARCH

We did not measure tHb‐mass and PV after paracentesis. We would advocate that future studies do so, plotting the change in PV (and weight) which occur over time. Future studies might also address the relationship between circulating albumin concentrations/oncotic pressure, ascitic volume, and PV.

There is no clinical cut off which defines “pathophysiologically low” tHb‐mass in the same way in which the WHO has defined a lower limit of [Hb] to define “anaemia.” Further research should define normative data for PV and tHb‐mass for healthy subjects across genders and races, and over wide age ranges.

Further work is required in order to generate and validate appropriate CO dosing regimens in CLD patients. These might consider baseline COHb%; actual body mass, ideal body mass, the contribution ascitic/edema mass to overall body mass and true body composition; more precise measures of frailty and fitness; and indicators that a low [Hb] might in fact be due to PV expansion. Studies might focus on repeat measures using a variation in doses in the same subject to measure both accuracy and reliability.

Studies should also be extended across different disease groups—and most especially in those among who plasma volume may be subject to pathological variation. In those with liver disease, trials might address the use of this methodology in guiding clinical intervention (such as diuretic treatment or packed red cell transfusion).

## 6 CONCLUSIONS

The oCOR method can be safely used to measure total hemoglobin mass in patients with chronic liver disease and ascites, without adjustment of blood sample timings. Further work might refine and validate appropriate dosing regimens.

## CONFLICT OF INTEREST

JP has received financial support from Siemens Healthcare Limited for consumables and hardware for research into the measurement of hemoglobin mass (2015–2018). JP was given consumables from Intersurgical UK Ltd (2015–2018); has received honoraria for speaking and/or travel expenses from Siemens, Vifor Pharma and Pharmocosmos and has received unrestricted grant funding from Pharmacosmos. JP is unaware of any direct or indirect conflict of interest with the contents of this paper or its related fields. SK has no conflicts of interest. JO has no conflicts of interest. WS is a managing partner of the company “Blood tec GmbH,” but he is unaware of any direct or indirect conflict of interest with the contents of this paper. HM consults for Google Deepmind on health technology and is on the Council of the UK Intensive Care Society but is unaware of any direct or indirect conflict of interest with the contents of this paper or its related fields. MG is the vice‐president of CPX International. He also serves on the medical advisor board of Sphere Medical Ltd and the board of EBPOM Community Interest Company, Medinspire Ltd and Oxygen Control Systems Ltd. He has received honoraria for speaking for and/or travel expenses from BOC Medical (Linde Group), Edwards Lifesciences and Cortex GmBH and unrestricted research support from Sphere Medical Ltd and Pharmacosmos Ltd. He leads the Fit‐4‐Surgery research collaboration and the Xtreme Everest oxygen research consortium, which has received unrestricted research grant funding from BOC Medical (Linde Group), Deltex Medical and Smiths Medical. MPWG was funded in part from the British Oxygen Company Chair of the Royal College of Anaesthetists awarded by the National Institute of Academic Anaesthesia. All funding was unrestricted. The funders had no role in study design, data collection and analysis, decision to publish or the preparation of the manuscript. This work was conducted at the Southampton NIHR Biomedical Research Centre with participants studied within the Southampton NIHR Clinical Research Facility. All authors are unaware of any direct or indirect conflict of interest with the contents of this paper or its related fields. No funders had any role in study design, data collection and analysis, decision to publish or the preparation of the manuscript.

## AUTHORS CONTRIBUTIONS

JP, JO, MW, MG, HM, and WS conceived the study. JP and SK conducted the study and collected the data. JP and HM drafted the manuscript. SK, JO, WS, MW, MG, and HM all contributed to the draft. JP produced the figures and tables. JP collated the article including references. The final version was read and approved by all authors.
