# Supplementary material for: Application of the optimized carbon monoxide rebreathing method for the measurement of total haemoglobin mass in chronic liver disease
Source: Physiol Rep. 2020 Mar 24;8(6):e14402. doi: 10.14814/phy2.14402 (PMC7090373; doi:10.14814/phy2.14402)
Supplement: Supplementary file 1 [file PHY2-8-e14402-s001.docx]

**Appendix 1- Detailed description of the technique used to measure total haemoglobin mass using the oCOR.**

Each participant was seated for 15 min to allow stabilization of PV, after which a mouthpiece connected them *via* a container of ‘soda lime’~10g (carbon dioxide scrubber) to a spirometer (Spico-CO Respirations-Applikator, Blood Tec, Germany) and a 3-litre anesthetic bag pre-filled with 100% oxygen. The patient exhaled to residual volume, breathed in the CO dose via the spirometer, held their breath for 10s, then continued normal breathing into the closed circuit via the spirometer for 1 min 50s. The participant then exhaled to residual volume, this exhaled volume being collected and analyzed to quantify the CO not absorbed into the bloodstream. Disconnected from the mouthpiece, participants fully exhaled to residual volume into a CO gas analyzer (Dräger Pac 7000, Drägerwerk AG & Co. KGaA, Germany) before and at 4 min after CO rebreathing, in order to determine the end-tidal CO concentration and thereby the amount of CO exhaled after disconnecting the patient from the spirometer, that will also have not been absorbed into the blood.

Prior to commencing the rebreathing technique, the investigators inserted an intravenous cannula into the subject’s upper limb. Venous blood samples were taken via Na-heparinised syringes (RAPIDLyte, Siemens Healthcare Diagnostics Inc, USA) before (at baseline) and at 6, 8, 10, 12, 15 and 20 minutes after administration of CO gas (Figure 1). In these, COHb% was determined using a laboratory blood gas analyser (Radiometer, ABL800 FLEX, Copenhagen, Denmark). As is standard for this technique, each sample was analysed three times within 1 hour of collection and the mean value used. The accuracy of the analyser has been evaluated elsewhere (Turner et al., 2014b), and it was subject to regular maintenance and quality control checks. Haematocrit and [Hb] values were measured using HemoCue (HemoCue AB, Radiometer, Sweden) and blood gas analyser (Radiometer, ABL800 FLEX, Copenhagen, Denmark) respectively.

**Appendix 2- Calculation of tHb-mass**

tHb-mass was calculated using a specifically design excel spreadsheet (Microsoft Excel

2011 for Apple Macintosh) using the formula:

tHb-mass (g) = K x MCO (ml) x 100 x (ΔCOHb% x 1.39)-1, where

K = barometric pressure x 760-1 x [1(0.003661 x temperature)]

1.39 = Hüfner’s number (constant) (ml CO x g Hb-1)

ΔCOHb% = difference between baseline COHb% and COHb% post CO administration (for the established method: average of 6- and 8-min COHb% values, for the extended method COHb% measured in min 8, 10, 12, 15, and 20)

MCO = COadm – (COsystem + lung (after disconnection) + COexhaled (after disconnection + CO diffused to myoglobin), where

MCO= the total amount of CO in the bloodstream

COadm = CO volume administered into the system

COsystem + lung (after disconnection) = CO concentration in spirometer x (spirometer volume + remaining volume in the lung after disconnection)

COexhaled (after disconnection) = end-tidal CO concentration x alveolar ventilation x time

CO volume diffused to myoglobin was calculated according to Prommer and Schmidt (Prommer and Schmidt, 2007). After starting the CO-inhalation procedure, 0.3% of the administered CO diffuses per min to myoglobin; e.g. in case of administration of 50ml CO the CO-loss to myoglobin is 1,05ml after 7min and 3.0ml after 20min.

Residual volume and alveolar ventilation were calculated using accepted international methods (European Respiratory Society, 1993). CO concentration is in parts per million (ppm).

Blood volume (BV), plasma volume (PV) and red cell volume (RCV) were calculated

from mean corpuscular haemoglobin concentration (MCHC), [Hb] and tHb-mass, as

below:

- BV (ml) = tHb-mass (g)/[Hb] (g^.^dl^-1^) • 100
- RCV (ml) = tHb-mass (g)/MCHC • 100
- PV (ml) = BV – RCV
